# Spheroidization of Alumina Powders for Additive Manufacturing Applications by DC Plasma Technology

**DOI:** 10.3390/molecules30030453

**Published:** 2025-01-21

**Authors:** Pierpaolo Iovane, Carmela Borriello, Giuseppe Pandolfi, Sabrina Portofino, Anna De Girolamo Del Mauro, Giuliano Sico, Loredana Tammaro, Nicola Fedele, Sergio Galvagno

**Affiliations:** 1 ENEA, Laboratory Smart Components and Systems for Sustainable Manufacturing, Department for Sustainability, Division Technologies and Materials for Sustainable Manufacturing Industry, 80055 Portici, Italy; carmela.borriello@enea.it (C.B.); giuseppe.pandolfi@enea.it (G.P.); sabrina.portofino@enea.it (S.P.); giuliano.sico@enea.it (G.S.); loredana.tammaro@enea.it (L.T.); sergio.galvagno@enea.it (S.G.); 2 ENEA, Innovative Devices Laboratory, Energy Technologies and Renewable Sources Department, Solar Photovoltaics Division, 80055 Portici, Italy; anna.degirolamo@enea.it; 3 ENEA, Laboratory Regenerative Circular Bioeconomy, Department for Sustainability, Division Sustainable Agrifood Systems, 80055 Portici, Italy; nicola.fedele@enea.it

**Keywords:** plasma, aluminum oxide, spheroidization, additive manufacturing, sintering

## Abstract

Alumina is the most widely used oxide ceramic, and its applications are widespread in engineering and in biomedical fields. Its spheroidization was performed by a prototypal direct current (DC) thermal plasma, which was designed and installed at ENEA, investigating surface morphology, particle size distribution, crystallinity, spheroidization, and reactivity. Features such as morphology and porosity significantly influence the flowability of the powder on the printer bed and, consequently, the density of the printed parts. It has been reported that spherical powder shape is highly recommended in additive manufacturing (AM) due to its superior flowability compared to other shapes whose interaction between powder particles results in poor flowability. In this paper, the spheroidization process of alumina powders using two different DC plasma powers and two kinds of secondary gas is reported. The average value of the circularity of the powders, after plasma treatment, has always been greater than or equal to 0.8 with the degree of the spheroidization over 90% at high power. The best process parameters of the thermal plasma were properly selected to produce spherical powders suitable for AM applications, and powders with high circularity were successfully obtained. Forming, debinding, and sintering tests were performed to verify the processability and the densification of produced powders, with good results in terms of density (97%).

## 1. Introduction

Traditional manufacturing methods for the production of ceramic parts include expensive processes, such as slip casting, pressing, or injection molding. These workflows are usually performed by specialized suppliers and require specific equipment and skills. Engineering and manufacturing teams that need few ceramic parts are forced to adapt to prices and processes that become cost-effective only at high volumes. Additionally, there are significant limitations on design: casting and molding workflows hinder design freedom and make it difficult to produce components with protrusions, internal channels, lattice structures, and other similar features. As has already happened for many other materials and applications, 3D printing could offer solutions to these challenges, making production extremely convenient thanks to rapid production of complex and customized parts [1]. The design flexibility of 3D printing brings new innovative possibilities to industries, requiring products with high thermal and chemical resistance, such as the aerospace and automotive industries but also in tooling, electrical, electronic, medical, and design industries [2,3]. For these applications, a small design change is enough to optimize air or fluid flow, and 3D printing enables the production of technical ceramic parts that can be used in extreme environments, finding the best design and creating complex features that would not be possible with casting or molding.

Alumina (Al_2_O_3_) is widely used in many of these sectors, thanks to its unique characteristics, such as high melting point, high mechanical strength, outstanding corrosion resistance and thermal insulating properties [4].

This material shows several polymorphic metastable phases, such as α, γ, κ, ρ, η, θ and χ, but α-Al_2_O_3_ (hexagonal structure) is the most thermodynamically stable [5,6,7] and the most used and known alumina, even if the applications of other polymorphs have been increasing over the past years [8]. The morphology and the properties of the powders influence the quality of the final product, but a spherical shape would make them ideal for demanding industrial applications using powder metallurgy, thermal spraying, and ceramic processing. Indeed, the uniform shape of spherical powders increases the contact area among the particles during sintering, promoting stronger particle bonding and improving material density and mechanical properties.

Plasma technology is one of the methods to produce nanoparticles and promote powder spheroidization [9,10,11], which normally requires high temperature and a rapid cooling system [12,13,14]. Radio frequency (RF) plasma is a consolidated technique to produce spheroized powders from irregular materials, while direct current (DC) plasma is a confirmed technology in plasma spray. Actually, this last system has higher efficiency and energy density than RF, features that turn out to be very attractive, especially when applied to the treatment of high melting point powders. Depending on process requirements, air, argon, oxygen, and nitrogen can be used to light the plasma, alone or in combination with secondary gases, such as hydrogen, oxygen, nitrogen, or helium, to raise the energy content, the thermal conductivity and to improve the flame conditions.

Under the same conditions, ceramics require higher energy than metals to be processed, due to their higher melting point and lower thermal conductivity [15].

Although alumina is one of the most important ceramics, a limited amount of works is found in the literature on alumina plasma spheroidization, most of them concerning RF-spheroidization.

Ye et al. (2004) [16] investigated the spheroidization of alumina powders (d_50_ = 24.5 µm) in Ar-H_2_ and Ar-N_2_ in RF plasma, obtaining spherical particles in both cases with different particle size distributions and crystal phases. The better results were obtained at about 28 kW in Ar–H_2_. The predicted and experimental data match each other very well under high feeding conditions, but the differences become larger as the feed rate decreases, suggesting an insight into the plasma–particle heat transfer model and the role of particle evaporation.

Zhen et al. (2017) [17] investigated the spheroidization of the alumina at different sizes (7 µm, 30 µm, and 75 µm) by RF plasma, varying different parameters of the process. The results in terms of spheroidization seem to be strongly dependent on the position of the injection probe, which influences the thermal histories of the treated particles. So, the spheroidization efficiency of synthesized powders (defined as the percentage of spherical particles out of the total particles) varies from <10% to nearly 99.9%, according to the parameters selected for the test.

Krishna et al. (2024) [18] examined the synthesis of spheroidal alumina powders (d_50_ = 84.5 µm) using RF plasma and analyzed their structural modifications for 3D printing applications.

Suresh et al. (2008) [8] spheroidized irregular micro-sized alumina powders (40–100 micron) by DC plasma with a conversion percentage of 71%; the main phase was α-Al_2_O_3_, with small amounts of γ-Al_2_O. The quenching rate of particles and the collection region (droplet-traversing distance after leaving the plasma flame) were found to be important parameters for the phase change.

Chaturvedi et al. (2014) [19] studied the spheroidization of alumina powder (15–38 µm) using a DC plasma torch, proving that the higher the plasma power, the higher the sphericity grade of the powders (even if a measurement of this parameter was not carried out) and the conversion to gamma-alumina. The conclusion was that in the process conditions, the powders produced were suitable to be used for thermal spray applications.

The shape factor and qualitative parameters of alumina spheroidization were sparse in the literature, so the effectiveness of the process is far from being understood, especially when using DC plasma, whose literature references are limited. In this frame, this paper investigates the characteristics of plasma-treated alumina powders using a DC thermal plasma. Depending on the selected size of the starting material, two different powers have been applied. Process and powder properties are characterized in terms of surface morphology, particle size distribution, crystallinity, spheroidization, and reactivity, giving new elements on the qualification of the process.

## 2. Results and Discussion

Tests were conducted both on raw and below 25 microns sieved calcinated alumina.

Based on the literature examination and preliminary experimental trials, two sets of process parameters have been selected for raw and <25 microns calcinated alumina. Among the different tests, conducted varying the plasma power in the range 16–35 kW, the gas flow rate (from 3 to 7 slpm for the carrier gas, from 35 to 60 slpm for the plasma gas), the type and the flow rate of the secondary gas (He and N_2_), two tests will be discussed, being representative of the most remarkable effects of the process on the produced powders; the adopted process conditions are summarized in Table 1.

The first test selected, for sieved powder, was conducted at low plasma power, 18 kW, and using He as secondary gas. Differently, in the second one, conducted on raw powder, plasma power was set at 28 kW, and N_2_ was employed as secondary gas.

During the process, the particles are injected into the plasma, where, entrained in the jet, they melt in flight, absorbing energy from the plasma, and achieve a spherical shape after cooling out the jet, due to the action of the superficial tension. This process is not complete when starting from non-homogeneous size powders and requires changes to the process parameters according to the particle dimensions. In fact, particles with different diameters experience different heating due to the size effect in the plasma flame [20]. Generally, if the energy is enough, the bigger particles melt and spheroidize, while the smaller particles absorb the energy required to evaporate and tend to form nanoparticles, often depositing on the surface of the bigger spherical particles.

**Figure 1:**
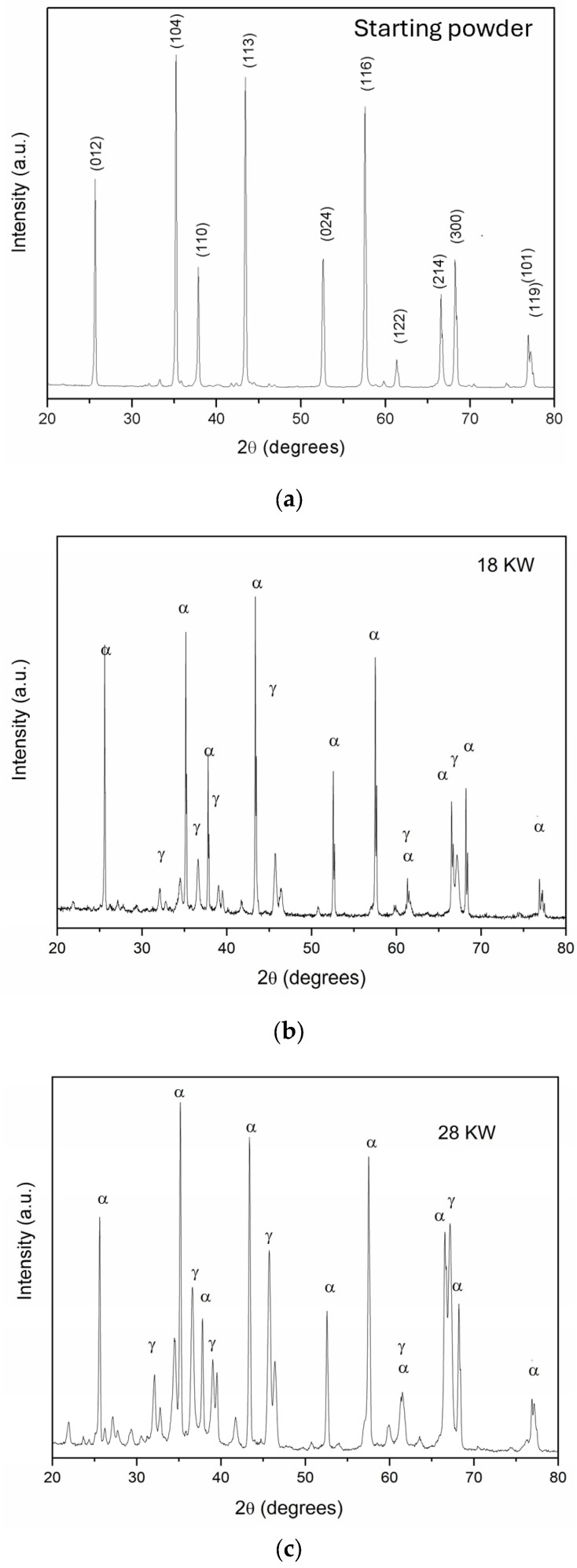
XRD patterns of alumina powders as received (**a**), after plasma treatment at low power (**b**), and high power (**c**).

The XRD patterns of the samples before and after the plasma treatment are reported in Figure 1.

The starting powder mainly exhibits α phase, which is the main phase after plasma as well, while the samples after treatment show other lower-intensity diffraction peaks. After plasma treatment, the solidification of liquid alumina does not necessarily lead to the formation of the most thermodynamically stable phase (*α*); the formation of metastable phases can compete with *α*-Al_2_O_3_ instead [20]. Actually, the nucleation of metastable phases of *γ*, *θ* and *δ* needs relatively lower critical free energy than *α* phase. Consequently, due to the rapid quenching of the particles melted and evaporated at high temperature after the spheroidization process, a portion of the particles could be composed of *γ*, *θ* and *δ* phases instead of *α* phase. The additional diffraction peaks in the spectra on the treated powders can be attributed to metastable phases, particularly *γ* phase. The intensity of *γ* phase increases at higher plasma powers (more energy for unit of mass) and at lower particle size distribution (minor energy needed to evaporate) [8,19,21].

**Figure 2:**
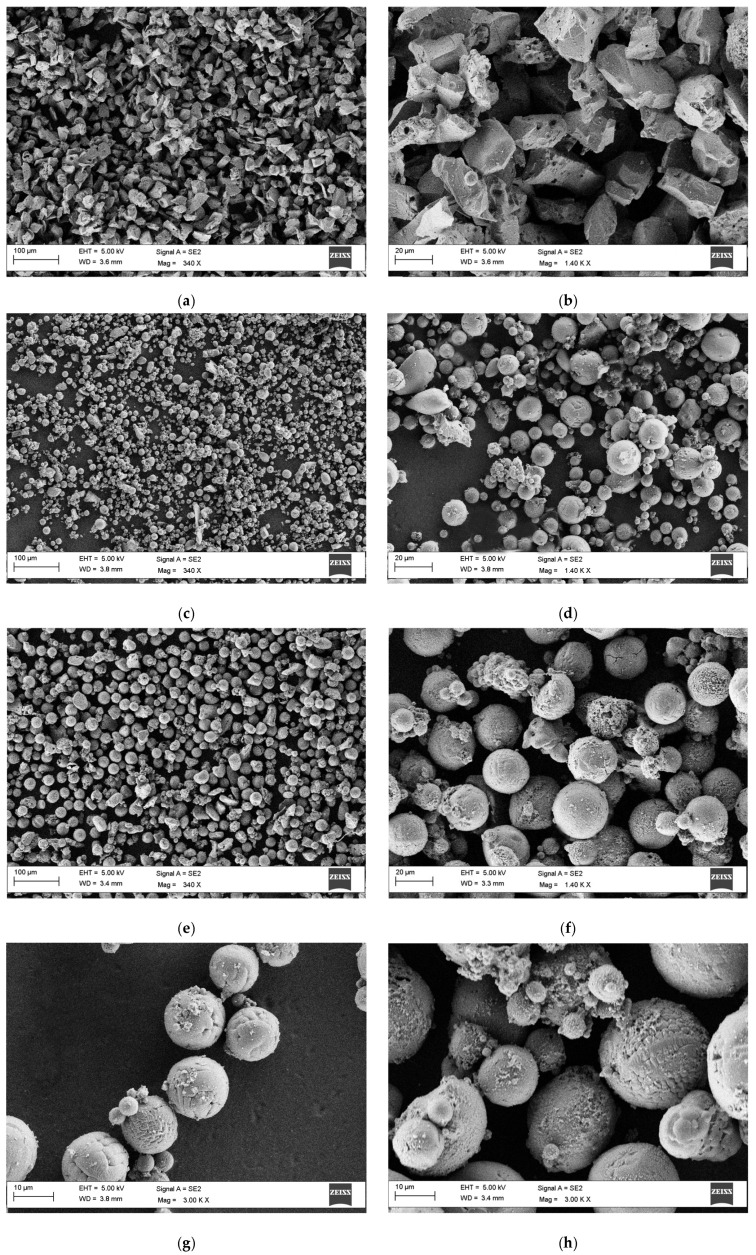
SEM images of alumina powders as received (**a**,**b**), after plasma treatment at low power (**c**,**d**) and high power (**e**,**f**); different magnification evidence cellular and dendritic morphology (**g**,**h**).

Figure 2 shows the SEM images of the alumina raw Figure 2a,b and after the plasma treatment, at low power Figure 2c,d and high power Figure 2e,f.

The calcined alumina (Figure 2a,b) presents irregular-shaped particles with the presence of both higher aggregates and smaller particles. After the plasma treatment, whatever the plasma powers, the samples look extensively spheroidized and nearly homogeneous.

With respect to the low power tests (Figure 2c,d), a power of 18 kW seems sufficient to achieve a good degree of spheroidization, but it was necessary to sieve the raw alumina under 25 µm. Due to the low dimensions, the powders after the test were collected just in the walls of the reactors and not in the tank.

Conversely, the tests at high power were conducted on the unsieved alumina, and the produced powders were collected both on the walls and in the reactor tank, then mixed and analyzed with respect to the morphology (Figure 2e,f): indeed, the SEM images show quite homogenous spherical powders with average dimensions higher than those presented at low power.

The images show spherical particles with small size flakes (nanoparticles) deposited on spheres; the surfaces also contain many bumps and deep trenches. These bumps could be regarded as the outer surface of many small grains, which are produced by relatively rapid cooling [11,22]. Due to its relatively high melting temperature (*Tm*_Al2O3_ = 2315 K), low boiling temperature (*Tb*_Al2O3_ = 3253 K) and a limited thermal conductivity (k_Al2O3_ 30 W/mK) [20], alumina has a high temperature gradient inside the particles and thus a high evaporation rate, which contributes to the formation of many fine particles on the surface of the big spherical ones. A relevant nanoparticle deposition on the external liquid surface of the particles, acting as nuclei, could locally change the cooling rate, which leads to the formation of dendritic/cellular structures. Indeed, several particles show dendritic or cellular morphology (Figure 2g,h). The increase in power generally brings about the formation of more nanoparticles, thus making purification of the powders necessary in view of further applications.

As previously mentioned, also circularity was evaluated from image analysis. The results show that the average circularity value is always higher than 0.8 (see Table 2), indicating an extensive spheroidization of the powders, whatever the raw powder processed.

**Figure 3:**
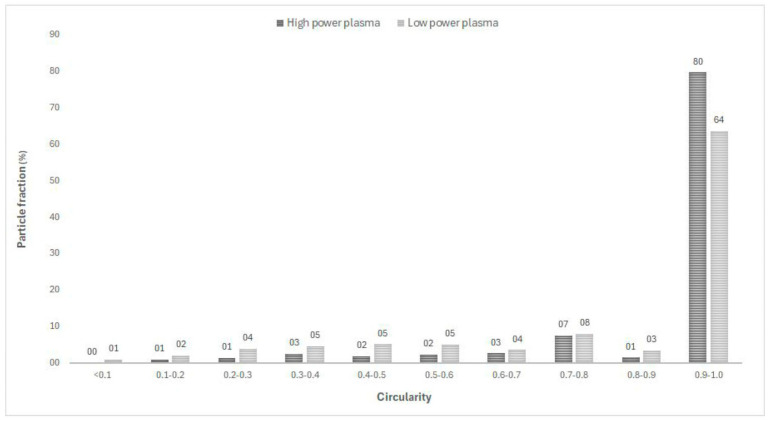
Circularity vs. particle fraction for low power and high power plasma tests.

The differences between the tests conducted at low and high plasma power are evident in Figure 3, where the particle fraction is reported versus the circularity of the powders: at high plasma power, nearly 80% of the powder have a circularity value in the range 0.9–1, while at low plasma power, this percentage decreases to 63%, revealing the extensive spheroidization obtainable at higher power.

The particle size distribution of the powders before and after the plasma treatment are reported in the following Table 3.

The spheroidized powder particle distribution, whatever the processing power, was narrower than that of the raw powder. This effect is equally confirmed by the SPAN, whose gap between the raw material and the processed powder is more obvious in the high-power than in the low-power test. Higher power leads to more rapid melting and evaporation of the surface of the particles so the average size of the spherical powder decreases [16,23].

The classification of the powders continues calculating HR and CI, and Table 4 shows the results on the produced powders.

HR and CI values are engineering indices for calculating the flowability of powders. A summary table of the standard values used for flowability based on Carr’s Index and Hausner’s Ratio is reported in the literature [24,25]. Relatively at HR, values less than 1.25 indicate good flowability (=20% CI), and values greater than 1.5 indicate low flowability (33% CI). For values between 1.25 and 1.5, it is necessary to add slip agents to improve flowability. The raw material has low flowability, which improves after subsequent plasma treatment. The best results have been achieved in the tests at high plasma power.

On these bases, the produced particles could potentially be considered a good starting material for ceramic additive manufacturing. The spherical shape of particles in the specific range could pack tightly the powder bed before sintering. This helps to achieve a homogeneous distribution of particles, a requirement for obtaining a uniform dispersion layer by layer into the bed to reach a high-performance 3D final product.

To explore the capacity of the powders to be sintered, useful for future exploitations, sintering tests have been performed by preparing pellets with a diameter of about 13 mm according to the procedure described above. The used process involves the Mixing/Forming/Debinding/Sintering cycle. Al_2_O_3_ starting powder, low- and high-power test powders were used, assuming only the alpha phase given the temperatures involved. Table 5 shows the density values measured after sintering.

The results show that, under the adopted process conditions, the plasma-treated powder densifies better than the starting powder. Collected results are in line with data reported in the literature [26]. Further tests are underway to improve the treatment conditions (pressing and sintering cycle) and to achieve higher densities.

## 3. Materials and Methods

A calcined alumina powder provided by 2B Minerals srl was used for the experimental tests (see Table 6 for the powders properties as reported in the datasheet).

**Figure 4:**
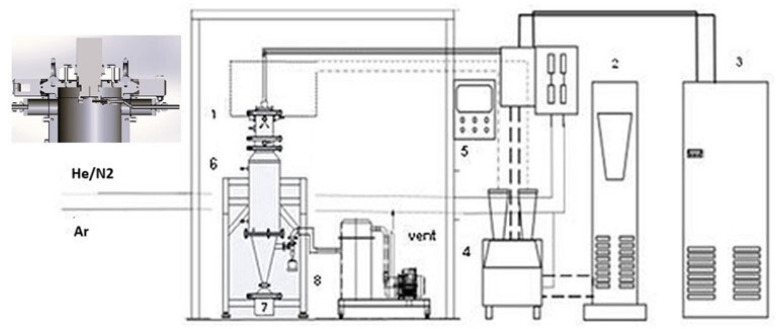
Plasma plant flow sheet with powder nozzle arrangement: (1) plasma torch, (2) power supply, (3) chiller, (4) powder feeder, (5) control unit, (6) reactor, (7) collection tank, (8) bag filter.

The raw powders were processed in a DC thermal plasma plant, which was designed and installed at the ENEA Research Centre of Portici (Italy) (Figure 4).

This system, already used for other experimental surveys and thoroughly described elsewhere [10], is composed of a DC non-transferred plasma torch, a power supply, a cyclone/bag filter, and a vacuum pump. The torch contains a standard tungsten cathode and a copper/tungsten 8mm anode nozzle, and it is placed on a cylindrical stainless-steel reactor, cooled with circulating cold water. The powders enter through a 2.4 mm nozzle (with an injection angle of 75°) directly into the plasma flame. At the bottom, a collection tank collects both the processed powders and the unreacted materials. Argon (Ar) was used as the main gas to light the plasma, and helium (He) or nitrogen (N_2_) were used as secondary gases to improve the plasma conditions. Argon was also used as a carrier gas for powder injection.

Due to its higher thermal conductivity, helium could enhance the heat transfer in plasma. On the other hand, replacing He with N_2_ increases plasma energy due to the contribution to the dissociation of the diatomic molecule; moreover, its safe handling and cost are much more advantageous than those of other gases. This choice allows us to work at higher power, reaching high energy with small quantities of secondary gas. These results are useful to process high melting point raw materials, where high powers are necessary to melt and then spheroidize them, thanks to the surface tension action.

Table 7 shows the comparison of the operating conditions with Ar-He and Ar-N_2_ without feeding, where the effects of the gas choice are evident. Indeed, with no N_2_, the power is 13.4 kW, and it does not change with 1 slm of N_2_, but it reaches 24.4 kW, thus almost doubling, at the highest tested flow.

Powders were sieved by a Retsch AS basic 200 vibrating sieving machine (Haan, Germany) and thoroughly characterized.

The information regarding the crystallinity and the structural phase of the powders were obtained by X-ray diffraction (XRD) analysis, by a X’Pert MPD diffractometer using nickel filtered Cu K *α* radiation in the range of 2θ  =  20°–80° with a 0.050° step width and a 5 s counting time for each step. The data were obtained by comparing the diffraction patterns of calcinated and plasma-treated powders with Al_2_O_3_ Joint Committee on Powder Diffraction Standards (JCPDS) cards: JCPDS No. 71-1123 for α-Al_2_O_3_ and JCPDS No. 10-0425 for γ-Al_2_O_3_.

Morphological analyses of the samples were carried out using a scanning electron microscope (SEM, Carl ZEISS LEO 1530 SMT GmbH, Oberkochen, Germany) equipped with a filament of tungsten at 4 kV and working under high vacuum conditions. The SEM pictures were used and further processed to measure the particles’ dimensions. Image processing and calculation were carried out in the image editor “ImageJ” (Software version 1.54g, NIH and LOCI, Madison, WI, USA)

The main parameters derived from the image analysis were circularity and spheroidization [27]. The circularity, calculated according to the following formula, is assumed as the main reference parameter of the shape factor [28]:

Circ = 4πA/P^2^ (1)

where P and A are the particle perimeter and area, respectively. The value varies from zero to 1, with a perfectly spherical particle having a circularity equal to 1. The degree of spheroidization is evaluated as the percentage of particles whose circularity is over 0.8 [29,30].

To determine the particle size distribution (PSD) of the materials, a Laser Diffraction MICROTAC MRB SYNC 3R (Montgomeryville, PA, USA) equipped with the dry dispersion TURBOSYNC system (Rawalpindi, Pakistan) was used. The results are expressed in d10, d50, and d90, where d90 indicates which 90% of the total volume of the material in the sample is contained, as for d50 and d10. An additional parameter used to quantify the distribution width and defined by Equation (2) is:

SPAN = (d90 − d10)/d50(2)

This parameter shows the distance between the 0% and 90% points, normalized with the midpoint; if the span is closer to 0, the PSD is more uniform and the size consistency is better, but it depends on the characteristics of the sample.

An important parameter for the classification of the powders is the flowability. For metal powders, it is evaluated according to a method based on Hall and Carney flowmeter. For ceramic-type powders, which are highly cohesive, methods based on density measurement are normally employed. The characterization of the alumina powders was carried out by measuring both the bulk density (BD) [31] and the tapped density (TD) [32]. Bulk density is the density (mass/volume) of the piled solid, including spaces occupied by voids between particles; tapped density is instead the maximum density of the piled powder, pressed without deforming the particles (voids between the particles reduced to a minimum). The measured values were used to calculate the Hausner’s ratio (HR) [33] and the Carr’s index (CI), defined, respectively, as:

HR = TD/BD(3)

CI = (TD − BD)/BD × 100(4)

HR from 1.00 to 1.34 means that the material has a higher and acceptable flowability, while a value greater than 1.60 is indicative of very poor flow properties [34,35].

For the sintering of the samples, a slurry was prepared by adding the powders, in quantities equal to 65% *w*/*w*, to deionized water containing PEG 200 (5% *w*/*w*) used as plasticizer and binder. After drying to remove the excess water, the slurries were subjected to forming by a SPECAC uniaxial press, operating at room temperature, equipped with a circular die with a diameter of 13 mm. The tablets were obtained by pressing for 30 min at 7 tons. All samples underwent a debinding process in a Nabertherm L 40/11 BO muffle, heating at 60 °C/h in air up to 450 °C and dwelling for 1 h. The sintering was conducted in Nabertherm LHT 08/18 L8 high temperature furnace, heating the samples in controlled atmosphere by flowing nitrogen (100 L/h). The used cycle involves a heating ramp up to 1400 °C at the rate of 300 °C/h, followed by a heating ramp up to 1700 °C at 180 °C/h and an isotherm of 1 h.

## 4. Conclusions

In this work, experimental spheroidization tests on Al_2_O_3_ powder were conducted using a prototypal DC thermal plasma plant developed at the ENEA. Two kinds of secondary gas, He and N_2_, have been used to improve energy exchange and content in plasma. Tests have shown that the particle size of raw materials plays an important role in determining process conditions. All other factors being equal, finer powders require less power to be processed. The best results were obtained by treating Al_2_O_3_ powders under the conditions shown in the paper, at 18 kW of plasma power and with He as a secondary gas for powders below 25 microns and at 28 kW of plasma power and N_2_ as a secondary gas for not sieved powders. The coarser powder showed a good degree of spheroidization after treatment; nevertheless, a large amount of nanometric fraction, together with a wider cellular and dendritic morphology on bigger spheres, was registered. Post-processing dust purification (leaching) is very important to obtain usable products. The average value of the circularity of the powders, a parameter used as shape factor, has always been greater than or equal to 0.8; in addition, the degree of spheroidization of the powders, a measure of the fraction of spherical powders produced, grows up to 97% at high power, indicating that the PS can be considered complete under the adopted experimental conditions.

After plasma the PSD of the samples showed a shaper distribution with respect to the starting powder.

Sintering tests have shown that powders produced by plasma can be considered for densification in AM or other manufacturing processes. Indeed, further tests are needed to achieve a more complete characterization of the powders before industrial applications. These results are particularly encouraging in view of the size of the experimental set-up, which could easily be scaled up for demonstration applications.

## Figures and Tables

**Table 1:** Comparison between two tests of plasma spheroidization of Al_2_O_3_ powder.

TEST	Power (kW)	Primary Gas (Slpm)	Secondary Gas (Slpm)	Carrier (Slpm)	Feeding Rate (g/h)
Low Power	18	Ar (40)	He (15)	Ar (3)	100
High Power	28	Ar (40)	N_2_ (3)	Ar (3)	100

**Table 2:** Powder characteristics (circularity and spheroidization).

TEST	Mean Circularity	Spheroidization (%)
Low Power-340x	0.83 ± 0.27	67.0
High Power-340x	0.92 ± 0.19	97.0

**Table 3:** PSD of alumina powders, before and after low- and high-power plasma.

TEST	Power	d10 (µm)	d50 (µm)	d90 (µm)	SPAN
Raw Material (d < 25 µm)	-	2.3	25.2	54.4	2.1
Low Power	18	2.9	15.4	32.2	1.9
Raw Material	-	4.3	79.7	178.4	2.2
High Power	28	20.6	41.1	92.1	1.7

**Table 4:** Powders characteristics (density, HR and CI).

TEST	BULK (g/cc)	TAPPED (g/cc)	HR	CI (%)
Raw Material	1.5	2.1	1.4	28.0
Low Power	1.3	1.6	1.2	23.0
High Power	1.4	1.6	1.1	14.0

**Table 5:** Sintering tests on Al_2_O_3_ pellets.

TEST	ρ_g_ (g/cm^3^)	ρ_g_/ρt (%)
Al_2_O_3_ (theoretical)	3.98	100
Raw material	3.74	94
High Power	3.82	96
Low Power	3.85	97

**Table 6:** Data sheet of alumina powder.

Analysis XRF		Physical Properties	
Al_2_O_3_	99.00%	Color	White
Na_2_O	0.35%	Morphology	Irregular
Fe_2_O_3_	0.02%		
SiO_2_	0.02%		
CaO	0.03%		

**Table 7:** Ar-He vs. Ar-N_2_ main operating conditions.

Ar (slm)	He (slm)	N_2_ (slm)	I (A)	V (Volt)	POTENZA (kW)
40	15	0	400	35	13.4
40	0	1	400	37	13.4
40	0	2	400	37	14.6
40	0	2	420	37	15.2
40	0	2	530	38	19.4
40	0	2	550	38	20.2
45	0	2	550	38	20.9
45	0	3	550	42	22.2
45	0	3	600	42	24.4

## Data Availability

The data that supports the findings of this study are available within the article.
